# The Influence of Cigarette Smoke Exposure on the Copper Concentration in the Serum Depending on the Use of Menopausal Hormone Therapy

**DOI:** 10.1155/2017/5732380

**Published:** 2017-08-14

**Authors:** Maria Jasińska-Starczewska, Iwona Szydłowska, Bożena Mroczek, Maria Laszczyńska, Dariusz Chlubek, Ewa Kemicer-Chmielewska, Kornel Chełstowski, Beata Karakiewicz, Sylwester Ciećwież, Andrzej Starczewski

**Affiliations:** ^1^Department of Public Health, Pomeranian Medical University in Szczecin, 48 Żołnierska Str., 71-210 Szczecin, Poland; ^2^Department of Gynecology, Endocrinology and Gynecological Oncology, Pomeranian Medical University in Szczecin, 2 Siedlecka Str., 72-010 Police, Poland; ^3^Department of Humanities in Medicine, Pomeranian Medical University in Szczecin, 11 Chłapowskiego Str., 70-103 Szczecin, Poland; ^4^Department of Histology and Developmental Biology, Pomeranian Medical University in Szczecin, 48 Żołnierska Str., 71-210 Szczecin, Poland; ^5^Department of Biochemistry and Medical Chemistry, Pomeranian Medical University in Szczecin, 72 Powstańców Wlkp. Str., 70-111 Szczecin, Poland; ^6^Department of Laboratory Medicine, Pomeranian Medical University in Szczecin, 72 Powstańców Wlkp. Str., 70-111 Szczecin, Poland

## Abstract

This study evaluated the effect of menopausal hormone therapy (MHT) on serum concentration of copper in postmenopausal women depending on passive or active exposure to tobacco smoke or lack thereof. The study included healthy postmenopausal women aged 42–69 years, who used (*n* = 76) or did not use (*n* = 76) MHT. Salivary cotinine and serum copper concentrations were determined in all the study subjects. Salivary cotinine exceeded 14 ng/ml in 14 women from the MHT group (18.5%) and in 16 controls (21.1%). Up to 41 (27%) study subjects had serum copper above the upper normal limit (1.17 mg/l). No correlation was found between salivary cotinine and serum copper in women with cotinine concentrations <14 ng/ml, and these two parameters correlated weakly in subjects with cotinine >14 ng/ml. Salivary concentration of cotinine increased with serum copper level in the MHT group, but not in the controls; smokers using MHT presented with significantly higher serum copper than nonsmokers. These findings imply that MHT does not affect serum concentration of copper in women who are not exposed to tobacco smoke. However, MHT seems to contribute to unfavorable increase in serum copper in passive and active smokers.

## 1. Introduction

Contemporary diet is getting more deficient in vitamins and microelements because of the consumption of highly processed food produced on an industrial scale [1, 2]. Also, environmental pollution, stress, and drug abuse affect the frequent occurrence of symptoms connected with deficiency of such biocomponents, such as magnesium, zinc, and selenium, and the excess of others, including copper and heavy metals [3, 4]. The effect of those changes on the health of perimenopausal women seems to be particularly interesting [5–7]. The manifestation of symptoms associated with fluctuations in these microelements' concentrations is quite nonspecific. It is connected with menopausal symptoms occurrence, which also causes worsening of the quality of life of these women. This is particularly observed as problems in the mental sphere and is manifested in the lack of concentration, nervousness, irritability, insomnia, fatigue, and exhaustion [8–12].

Copper is a microelement necessary for the proper functioning of the body. It acts as a cofactor in ca. 30 copper-dependent enzymatic systems, such as cytochrome C oxidase, dopamine, hydroxylase, tyrosinase, and cytotoxic and extracellular superoxide dismutase [13, 14]. In humans, this element plays a protective role in leukopenia, bone demineralization, arterial rigidity, and neural fiber demyelination in relation to the activity of copper-dependent enzymes [14]. Copper causes man to be more dynamic and vigorous, experiencing a tide of optimism. Some have suggested that copper is also responsible for falling in love, enlightenment, and euphoria [6, 15]. However, this microelement is toxic in excessive amounts. Higher concentration of copper causes problems with the function of the kidneys, liver, and cardiac muscle and damages the central nervous system with manifestation of mental symptoms [16, 17].

Cotinine is a compound, a derivative of pyrrolidine, and a metabolite of nicotine. The half-life of this substance in the body is approx. 20–40 hours and it can be detected at very low concentrations, down to 0.1 ng/ml. Cotinine concentration ≤ 14 ng/ml is the criterion for active smoking [3]. Lower values indicate passive exposure to tobacco smoke or high contamination of the environment. Assessment the concentration of cotinine is considered as an objective biomarker of exposure to tobacco smoke [18, 19]. Currently, it is believed that cotinine may play an important role in inflammation and some neurodegeneration. It has been proven that it binds to nicotinic cholinergic receptors in the brain. Researches on bovine adrenal chromaffin cells showed the influence of cotinine on the activity of C protein and the calcium concentration in these cells [20].

Many previous studies demonstrated that smoking may influence serum concentrations of calcium and selenium. In contrast, very little is known about the impact of smoking on metabolism of other microelements. However, published evidence suggests that exposure to environmental pollutants present in the region of copper mines, and smoking, may result in a decrease in menopausal age [7, 21, 22].

The aim of this study was to evaluate the effect of menopausal hormone therapy (MHT) on the concentration of copper in the serum of postmenopausal women depending on smoking or passive smoke exposure or lack of that exposure.

## 2. Materials and Methods

Participation in the study was voluntary. Women willing to participate in the project were recruited in family physician and gynecology practices, after familiarizing themselves with the invitation form and additional information about the profile of laboratory tests. All study subjects provided their written informed consent to participate in the project.

The study included 152 healthy women (aged 42–69 years) with intact ovaries and uteruses, who were at least one year after menopause. Qualification to the study was based on an interview. The criterion for exclusion from the study was a history of chronic diseases such as diabetes, neoplastic disease, liver disease, kidney failure, hypertension, or thyroid disease. The women were divided into two groups: those using transdermal estrogen-progestin menopausal hormone therapy in the form of patches (Group I) and controls who did not use this form of hormone therapy (Group II). In each group, there were 76 women.

Selected sociometric data were collected with a questionnaire developed by the authors. The list of analyzed sociometric variables included age, place of residence, education, marital status, BMI, and professional activity. In all subjects, concentrations of cotinine in saliva and copper in serum were assessed to determine whether the woman was exposed (passive or active) to tobacco smoke. Serum was obtained from whole blood (5 ml) using a vacuum system. Following centrifugation (5,000 rpm), the sera were frozen at −80°C and dispatched to the laboratory. Serum concentration of copper was determined by means of absorption spectroscopy with the flame technique, using PU 9100X device from Philips. Appropriately diluted serum samples were passed through the flame. The samples were diluted 80-fold with lanthanum dissolved in hydrochloric acid. The results were read at a 285.2 nm wavelength. Concentrations of copper were read from a standard curve. The tests were carried out at the Biochemistry Unit, Department of Medicinal Biochemistry and Chemistry, Pomeranian Medical University in Szczecin.

Saliva samples were collected to double Salivette-type tubes, after placing citric acid-impregnated adsorption paper in the oral cavity to stimulate saliva production. After 5–10 min, the adsorption paper was centrifuged with a 0.7 to 1.5 ml yield of saliva ultrafiltrate. Salivary concentration of cotinine was determined with a liquid chromatograph, HPLC Waters 2650, with a 150 × 21 mm analytical column XTerra MS-18, tandem mass spectrophotometer Quattro Micro, and data processing and acquisition system MassLynx v. 4.0. The tests were conducted at the Research Laboratory for Organic Environmental Pollutants, Department of Chemical Safety, Institute of Occupational Medicine in Łódź.

Protocol of the study was approved by the Local Bioethics Committee at Pomeranian Medical University in Szczecin.

Statistical analysis was conducted with Statistica v. 13.1 PL (StatSoft, Tulsa, USA). Statistical characteristics of continuous variables were presented as arithmetic means (*x*) and their standard deviations (SD), medians, and minimum and maximum values. Distributions of qualitative variables were shown as numbers and corresponding percentages (fractions). Normal distributions of copper and cotinine concentrations were verified with Shapiro-Wilk test; concentrations of cotinine were subjected to logarithmic transformation. Mean values of selected parameters were compared with Student's *t*-test. Correlations between concentrations of cotinine and copper were expressed with Spearman's coefficients of rank correlation (*r*_*s*_), along with their *p* value. The effect of interaction between MHT status (users versus nonusers) and cotinine level (cut-off value: 14.0 ng/ml) on the serum concentration of copper was verified using analysis of variance (ANOVA), with Bonferroni post hoc test. Results of all statistical tests were considered significant at *p* ≤ 0.05.

## 3. Results

Detailed characteristics of the study subjects are presented in Table 1. MHT users and nonusers did not differ significantly in terms of any of the analyzed sociometric parameters, including age and time elapsed since menopause. Only 15 women (8 from the MHT group and 7 from the non-MHT group) had menopause before 46 years of age. A survey demonstrated that 30 women (19.7%), 15 (19%) per each group, were smokers. Furthermore, the study groups did not differ in the proportion of coffee drinkers (71% versus 67%) and alcohol drinkers (12% versus 13%).

Salivary concentration of cotinine >14 ng/ml, a marker of heavy exposure to tobacco smoke, was found in 14 women (18.4%) from the MHT group and in 16 women (21.1%) from the non-MHT group. A total of 62 women from Group I (81.5%) and 60 from Group II (78.9%) had salivary concentrations of cotinine <14 ng/ml.

Up to 41 study subjects (27%) had higher levels of copper than the preferred range (0.8–1.6 mg/l).

**Figure 1:**
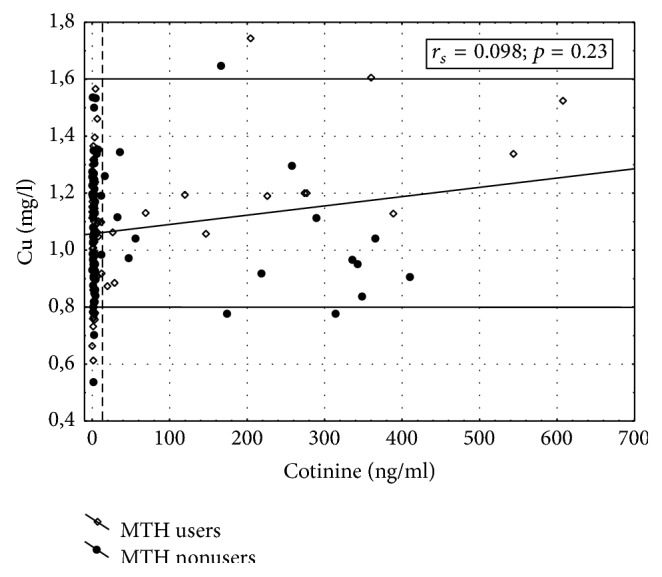
Correlation between serum concentration of copper and salivary level of cotinine in MHT users and nonusers (Groups I and II). The vertical line represents salivary cotinine level of 14.0 ng/ml; values ≥14.0 ng/ml correspond to active smoking. Thick lines on this and subsequent graphs represent recommended serum concentrations of copper.

Associations between serum concentrations of copper and salivary levels of cotinine in MHT users and nonusers (Groups I and II) are shown in Figure 1. No statistically significant correlation was found between salivary cotinine and serum copper in all study subjects. Moreover, no significant correlation (*r*_*s*_ = 0.039, *p* = 0.672) between these two parameters was observed in subjects who presented with low salivary levels of cotinine (<14.0 ng/ml). The percentage of participants with low copper levels is very puzzling and requires further consideration. The latter relationship was not presented graphically owing to the journal's limits in the number of figures. High salivary concentrations of cotinine found in some individual cases suggest that these women have smoked a cigarette shortly before testing.

**Figure 2:**
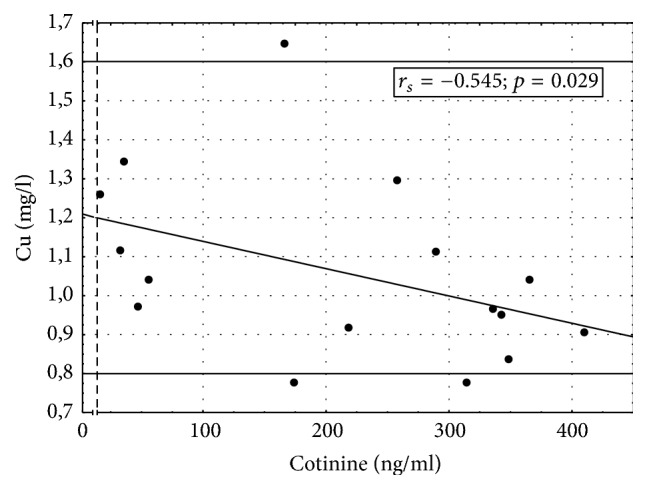
Correlation between serum concentration of copper and salivary level of cotinine in MHT nonusers exposed to tobacco smoke.

**Figure 3:**
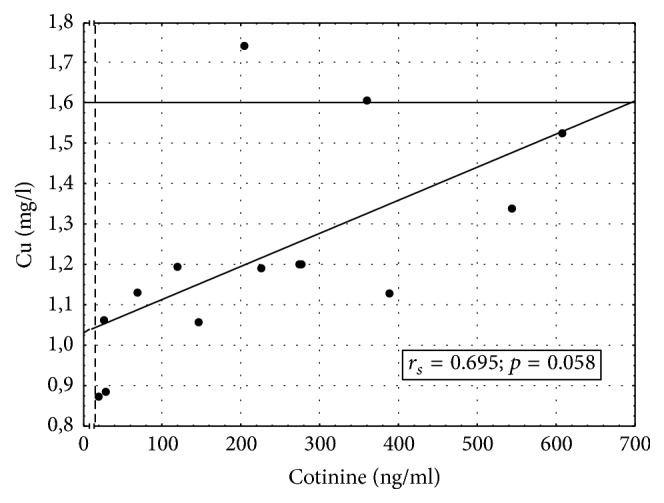
Correlation between serum concentration of copper and salivary level of cotinine in MHT users exposed to tobacco smoke.

Associations between serum concentrations of copper and salivary levels of cotinine in women using/not using MHT and being exposed to tobacco smoke are shown in Figures 2 and 3.  Figure 2 documents a significant inverse correlation between salivary cotinine and serum copper in women who were exposed to tobacco smoke (salivary cotinine ≥ 14 ng/ml) but did not use MHT. In smokers (i.e., women with salivary cotinine ≥ 14 ng/ml) who used MHT, an increase in salivary concentration of cotinine correlated significantly with an increase in the serum level of copper.

**Figure 4:**
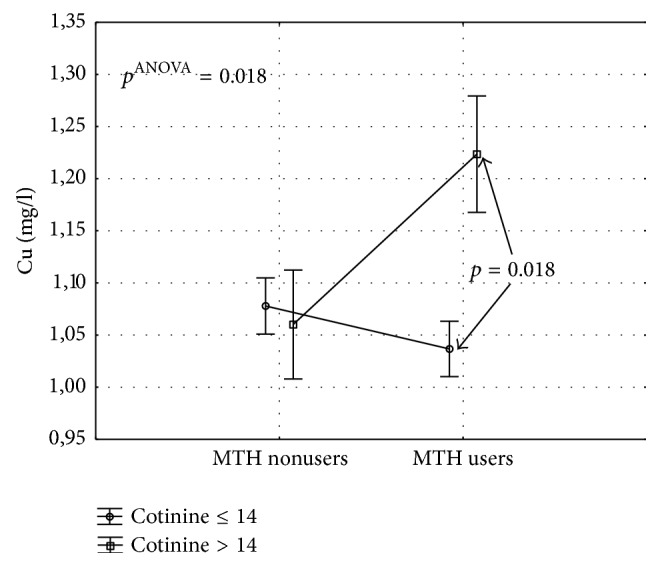
Effects of MHT use (I) or lack thereof (II) and exposure to tobacco smoke (salivary cotinine < 14 ng/ml) or lack thereof (salivary cotinine ≥ 14 ng/ml) on serum concentration of copper; results of ANOVA. *p*: statistical significance in post hoc Bonferroni test.

The results of ANOVA analyzing the effects of MHT use (I) or lack thereof (II) and exposure to tobacco smoke (serum cotinine ≥ 14 ng/ml) or lack thereof (serum cotinine < 14 ng/ml) on serum concentration of copper are presented in Figure 4. As shown in the figure, there was a significant interaction between MHT use and exposure to tobacco smoke (*p*^ANOVA^ = 0.018). Post hoc tests revealed that women who used MHT and were exposed to tobacco smoke presented with significantly higher serum concentrations of copper than other study subjects.

## 4. Discussion

At the forefront of the improper lifestyle in postmenopausal women is the lack of physical activity [21]. Another problem is inadequate diet, and not only energy balance but also the quality of food products is important. Supplementation of microelements and vitamins has great significance. Also, overusing drugs and environmental pollution significantly reduce absorption of some microelements and enhance absorption of others. These components are predictors for nonspecific symptoms of deficiency or excess of various bioelements, which overlap with menopausal symptoms, characteristic in this period of life. Currently, an important therapeutic tool to improve quality of life of these women is the use of MHT [7, 22]. It is proven to have a positive impact not only on reducing menopausal symptoms, but also for prevention of many diseases [6, 18, 23]. However, based on recent reports, it seems important to study the impact of concentrations of microelements as indicators, which increases quality of life during menopause. A healthy lifestyle can contribute to a decrease in the intensity of menopausal symptoms and thus improve the professional and psychosocial functioning of these women [8, 24].

There is a proven relationship between overweight or obesity and self-reported poor quality of life. The authors indicate a connection between decreased magnesium concentrations and elevated copper concentrations in women with a higher BMI [25, 26]. Concentrations of copper and zinc correlate with each other; dietary deficiency of zinc results in enhanced absorption of copper in the alimentary tract. An average zinc-to-copper ratio in food is 1 : 10, while the presence of vitamin C inhibits the absorption of copper. This is very important due to the fact that copper taken and/or absorbed in excess (environmental pollution, including the risk of exposure to tobacco smoke, intense supplementation) is a toxic element [27, 28]. Our hereby presented findings suggest that, in women on MHT, active smoking or passive exposure to tobacco smoke may be associated with serum accumulation of copper.

Vitoux et al. [19] believe that determination of copper concentration in erythrocytes requires further studies, because it is still very difficult to assess its deficiency or excess. In the case of Wilson's disease, the concentration of copper inside the erythrocyte should be considered with extreme caution. According to recent data, it was shown that the early appearance of disease symptoms such as hemolytic crisis can be connected with oxidative stress and release of free copper from the red blood cells [19].

Ugarte et al. [29] noticed the association of copper concentration with neuropathological symptoms and perhaps impaired renal function. Squitti et al. [6] emphasize the relationship between zinc supplementation greater than 150 mg/day and copper concentration in the blood and brain. These results support the thesis of a possible diet-dependent ground of some mental disorders like dementia [6].

Lopes et al. [30] reported that serum copper concentration was higher in women than in men and decreased with age; however, these authors did not evaluate the impact of MHT on copper concentration in female subjects. Unfortunately, we were unable to determine sex-specific differences in serum copper as our study included solely women.

Our study showed that 27% of women had levels of copper higher than the preferred range. There may be many reasons for this state. The most important appears to be environmental pollution including water. To the best of our knowledge, this issue has not been addressed in postmenopausal women thus far. The District Sanitary Inspector in Szczecin provided information that, in the six months preceding our study, the concentration of copper in samples intended for human use did not exceed 0.050 mg/l, with maximum acceptable norm of copper concentration in waterworks in Poland of 2 mg/l. This would indicate that the source of copper in the examined group of women is not tap water. Hence, the origin from food and air must be taken into consideration. In the air, one of the factors can be interactive influence of cigarette smoke, without behavioral differentiation like active or passive smoking, on the increase of this bioelement [20, 31]. In this study, passive or active exposure to tobacco smoke was associated with an insignificant increase in serum concentration of copper in postmenopausal women; however, we found a significant correlation between salivary concentration of cotinine and serum copper level in women who used MHT. Very interesting is the fact that menopausal hormone therapy does not affect the serum copper concentration in women not exposed to tobacco smoke, which has not been studied up to now. Theoretically, MHT should increase serum concentration of copper since, as shown by Lopes et al. [32], estrogens stimulate synthesis of ceruloplasmin, a carrier protein for this element. Most observed alterations in plasma concentration of copper are associated with changes in ceruloplasmin levels, determined by factors such as age, sex, hormone replacement therapy, pregnancy, and inflammation [33].

Koniarek et al. [31] confirmed in their reports that exposure to the toxic copper has a bad effect on the women's health. They found that women living in Poland in the Copper District are more exposed to the negative impact of environmental pollution. In addition, cigarette smoking intensifies this action. These authors observed also a synergy of these two harmful factors. Since research has been conducted on nearly 10 thousand women from the Copper District and Wrocław, results can be considered as confirmed. It is worth noticing that menopause, in women from the Copper District, was about one and a half to almost two years earlier than women inhabitants of Wrocław [31].

Significantly higher copper concentrations were found in the serum, but not in the hair, of women with osteoporosis [34]. However, these findings were not confirmed in another study [35], in which serum concentration of copper did not modulate the risk of osteoporosis.

Measurement of serum cotinine may be useful in assessing the health condition and health risks in women. Interaction with nicotine in the body may contribute inter alia to lower activity of liver enzymes and a decrease in blood concentration of catecholamines. The authors report also the influence of cotinine on the course of many diseases, from a positive like in ulcerative colitis to evidently unfavorable like in the case of Crohn's disease or breast cancer [36].

Rzymski et al. [37] analyzed concentrations of many elements in the endometrium and polyps from female reproductive tract, depending on subjects' habits. Cigarette smoking contributed to an increase in the concentrations of cadmium, lead, and copper in both normal and polypous endometrium.

MHT may interfere with the activity of CYP2A6, the main enzyme responsible for the oxidation of nicotine and cotinine, metabolism of drugs, alkaloids, and carcinogenic substances, and cholesterol synthesis [38]. Therefore, estrogens may compete with nicotine, disrupting its degradation, whereas hypoestrogenism may promote copper metabolism. Our study demonstrated a correlation between salivary cotinine and serum copper and showed that this relationship may be modulated by MHT use. In this study, women who were active smokers (as confirmed objectively based on salivary cotinine >14 ng/ml) and used MHT presented also with elevated serum concentrations of copper. Thus, this problem definitely needs to be addressed in future studies.

## 5. Conclusions

MHT does not affect serum concentration of copper in women who are not exposed to tobacco smoke. However, MHT may contribute to an unfavorable increase in serum concentration of copper in smokers.

## Figures and Tables

**Table 1:** General characteristics of the study subjects (*n* = 152); comparison of MHT users (*n* = 76) and MHT nonusers (*n* = 76).

Parameter	Whole group					MTH users	MTH nonusers	*p* ^#^
	*x*	SD	Min.	Max.	Med.	*x* ± SD	*x* ± SD	
Age (years)	55.9	5.1	42	80	55	55.9 ± 4.5	56.0 ± 5.6	NS
BMI (kg/m^2^)	26.7	4.2	19.0	43.0	25.7	26.3 ± 3.9	27.1 ± 4.4	NS
Menopause^*∗*^ (years)	50.1	4.1	39.0	65.0	50.0	49.8 ± 3.9	50.3 ± 4.2	NS
Cotinine (ng/ml)	46.3	113.9	0.1	607.8	2.66	45.7 ± 119.8	46.9 ± 106.4	NS^&^
Copper (mg/l)	1.07	0.21	0.54	1.74	1.06	1.07 ± 0.22	1.07 ± 0.21	NS

^*∗*^Mean age at menopause; ^#^Student's *t*-test; ^&^after logarithmic transformation.
